# Narratives of childless widows: exploring the lived experiences and well-being of childless widows in rural Nigeria

**DOI:** 10.1080/17482631.2020.1713657

**Published:** 2020-01-10

**Authors:** Dorothy I. Ugwu, Charles T. Orjiakor, Leonard I. Ugwu, Chucks E. Ezedum, Oliver R. Ngwoke, Comfort Ezebuilo

**Affiliations:** a Department of Human Kinetics and Health Education, University of Nigeria, Nsukka, Nigeria; b Department of Psychology, University of Nigeria, Nsukka, Nigeria; c Health Policy Research Group, Department of Pharmacology and Therapeutics, College of Medicine, University of Nigeria, Enugu, Nigeria; d Department of Linguistics, Igbo and Other Nigerian Languages, University of Nigeria, Nsukka, Nigeria

**Keywords:** Childless, distress, lived experience, low-to-middle-income, narrative, widow

## Abstract

**Background**: Widows are socioeconomically disadvantaged, especially in low resource regions. Childless widows are a group whose plight may be worse given sociocultural circumstances. In the current study, we examined the lived experiences of childless widows living in remote Nigeria, highlighting this group as being in critical need for social interventions.

**Method**: Childless widows (n = 11) in rural settings in South East Nigeria were interviewed. Narrative analysis was used in navigating the lived experiences of the widows.

**Results**: Extreme distress, ostracism, stigma, and traumatic experiences were common in the narratives of the widows. However, childlessness was at the core of their distress. Treated as outcasts, the widows resigned to God, though some were scarcely allowed to play supportive roles among relatives. As social welfare packages are almost non-existent in this region, religious groups often played supportive roles.

**Conclusion**: Legislation protecting widows are good but may not be sufficient if it does not translate to improved wellbeing/welfare for widows. Childless widows, especially those in rural areas, are especially vulnerable as they face peculiar deprivation and psychological distress arising from cultural/social realities. Recognising the limited resources in low income countries, mobilisation of local structures and resources to educate and monitor local communities are important.

## Introduction

Conservatively, there are an estimated 250 million widows in the world, many of who experience peculiar difficulties and deprivations that impact their livelihood, health and well-being (The Loomba Foundation, 2015; United Nations, 2019). Higher rates of physical illnesses and mortality have been reported amongst widows compared to their married counterparts (Anderson & Ray, 2018; Nalungwe, 2009). Also, severe psychological distress in the nature of loneliness, depression, obsessive thoughts, restlessness, insomnia, somatic complaints, hallucinations, and poor mental well-being have been reported among widows (Nalungwe, 2009). However, the experience of widowhood is not homogenous globally (Djuikom & van de Walle, 2018). Widowhood literature in low- and-middle-income countries and especially in the sub-Saharan region often feature socioeconomic challenges as well as cultural norms that make widowhood more traumatic in the region (United Nations, 2000).

The prevailing experiences of widows are determined by the social norms concerning widows in their locations (Milazzo & van de Walle, 2018). Having child(ren) is socially desirable in many parts of Africa (Muomah, 2010), and children may lessen the burden of widowhood. Children are sometimes the connection between a widow and the husband’s relatives as children often have inheritance rights to their fathers’ property (Milazzo & van de Walle, 2018). A pertinent question here is: what about widows who do not have children? In this paper, we identified childless widows (in rural South East Nigeria) to be a section of widows who may suffer intense hardship arising from their unique cultural backgrounds, where childlessness may intensify the pangs of widowhood. We hope to raise awareness on this specific group of widows by highlighting their lived experiences and how health and well-being are affected based on their stories.

Wanting children in marriage but not being able to procreate, especially in Africa, is a major source of psychological distress (Osayuki, Owofio, & Osondi, 2015). Societal deep-rooted stigma on childlessness in Africa exacerbates stress and depression in involuntary childless (Lisle, 1996). Childless widows are a group of widows that may endure protracted impact of widowhood in Africa given cultural values placed on having children (Matthias, 2015). For childless widows, not having children could worsen their plight as there is no hope for generational interdependence especially as the women age. Some traditional practices such as proscribing women from inheritance worsen the lot of women/widows in these settings leaving women alienated and in extreme poverty.

African widows face more discrimination and hardship and also have poorer well-being compared to their married counterparts (Anderson & Ray, 2018; Milazzo & van de Walle, 2018). Widows in rural areas of Nigeria have been described as the most vulnerable and marginalized group, given the ill-treatments they receive (Eboiyehi, 2013; Milazzo & van de Walle, 2018). There are wide reports of adverse widowhood practices in Nigeria attracting many not-for-profit and religious institutions to intervene in the plight of widows in the region (Milazzo & van de Walle, 2018). In rural Nigeria, cultural widowhood practices offer little support and often subject widows to extended trauma (Eboh & Boye, 2005; Ihekwuaba & Amasiatu, 2016). Among the Igbo ethnic group in South East Nigeria who are largely Christians, inhumane widowhood cultural practices are popular, and are worsened by social and religious norms (Milazzo & van de Walle, 2018). In the northern part of Nigeria, where Islam is the dominant religion, Muslim widows may not face so much distress as the dominant religion encourages polygamous family system and re-marrying which mean that widows in the region are easily integrated back to their communities (Milazzo & van de Walle, 2018).

Though there are government laws in the region prohibiting inhumane practices against widows, inhumane practices against widows persist. Some inhumane widowhood practices common in South East Nigeria are highlighted in some state laws. In 2001, legislation in Enugu State, South East Nigeria enacted laws outlawing the compulsion of widows to perform some practices: “shaving hair on the head or any other part of the body; sleeping either alone or on same bed or be locked in a room with corpse of the husband; preventing widows from receiving condolence visits from sympathizers during the period of mourning; compelling widows to remarry a relative of the late husband; sitting on the floor or be naked during any period of the husband’s burial rite; drinking the water used in washing the corpse of the husband; weeping and wailing loudly at intervals at any time after the death of the husband except at one’s own volition or involuntary action; remaining in confinement after the death of the husband for a given period; vacating the matrimonial home; or indeed to do any other thing which contravenes the fundamental rights entrenched in the constitution or is degrading to the person.” (Adapted from Matthias, 2015). Despite legislative pressures, some of these widowhood practices are still actively enforced by sociocultural norms. There are often frictions between cultural practices and state policies/laws, as well as human rights (Katiuzhinsky & Okech, 2012) which obstruct policy implementation. The lack of resources in low-resource regions adds to the difficulty in enforcing laws and policies especially in rural areas, giving room for abhorrent cultural practices to thrive. These conditions prolong and intensify the traumatic experiences of widows.

Antithetically, African cultures are popular for the strong support emanating from its extended family structures. The extended family has been referred to as a traditional social security system that caters for the vulnerable, poor, and sick (Foster, 2000). However, this traditional system is fast depleting, because of social changes arising from labour migration, demographic changes, urbanization and increased westernization (Foster, 2000). The absence of a state funded social welfare scheme in the region suggests more vulnerability and hardship for widows. Researchers need to probe into the plight of widows in different settings and contexts to follow up with the widowhood-deprivation links globally (Lloyd-Sherlock, Corso, & Minicuci, 2015). There are implications for policy following identification of the origin of distresses troubling African widows. Milazzo and van de Walle (2018) distinguish between policies targeting institutional shortcomings and those targeting familiar development among widows.

Although existing literature discusses extensively the plight of widows in general, there is paucity of literature regarding childless widows. The aim of the current study was to examine the lived experiences of being a childless widow living in remote Nigeria. The dearth of studies on the traumatic experiences and their impacts on childless widows’ well-being in the developed and developing countries presents a yawning gap in the widowhood literature. Studies are thus needed to understand the traumatic experiences of childless widows, especially those living in the rural areas in Nigeria.

## Method

### Design

Considering the nature of the participants in focus, we chose narrative analysis as a preferred approach in exploring and interpreting the experiences of childless widows. Narratives entail knowing and communicating personal experiences through stories. (Personal) narratives have been identified to be fit in examining the experiences of the marginalized and oppressed (see Langellier, 2001). Narratives include diverse approaches that include the use of entire life story or the use of brief specific stories about specific people or places (Riessman, 2005). We prefer to use personal narratives that draw from brief specific accounts of people’s lives in context. Additionally, we adopt a constructionist stance in executing the current study. The constructionist approach emphasizes that meaning and experience are socially formed rather than arising from individuals, therefore highlighting the socio-cultural contexts and structures that underlie individual accounts (Braun & Clarke, 2006; Burr, 1995).

### Data collection

A combination of purposive and snowball sampling technique was used in identifying the participants. One of the authors (DU) had experience working with charity organizations on the welfare of widows and had considerable insight on how to locate the widows as well as some of their experiences. After consulting with some of the not- for- profit groups, a study protocol was developed including an interview guide. Participants (n = 11) were identified using snowball sampling. Authors DU and CO conducted the interviews. Participants were interviewed on their experiences as widows without children. Interviews were kept open ended and lasted for an average of 40 minutes for each participant. Participants were allowed to talk with minimal interruption. Probes and prompts were used to elicit deeper narrations and information from the widows [e.g., tell me more about the people living around]. Interviews were recorded with an audio recorder and later transcribed verbatim. As most of the participants used the Igbo language in their discussion, the last author CE, who is an Igbo linguist, translated and transcribed the interview protocol and the transcripts. In order to relieve the burden of trauma memory reactivation, the researchers debriefed and offered free brief counselling sessions for the participating widows.

### Participants

Eleven childless widows from a rural region of Enugu State, South East Nigeria were purposively chosen to participate in the study. They were aged between 46 and 85. One widow was blind and lived with her relatives. All participants had been married according to the traditional rites common in the culture, and their husbands were deceased. Two of the women lived alone in traditional huts in very remote areas and had no close relatives living with them. However, they are occasionally visited by members of charity organizations. Four others lived with their relatives. These women received no pensions or welfare benefits of any kind from the government. Table I shows some characteristics of the participants.

**Table I.** Demographic and few defining characteristics of participants

	Pseudonym	Age	Other defining characteristics	Residence characteristics
1	Nwakego	90	Frail and weak, unable to do farm work	Alone, away from relatives, depends on charity
2	Mgbeke	86	Frail and weak, unable to do farm work but prepares her meals	Parents’ homestead but away from relatives, depends on charity
3	Chinasa	67	Subsistence farmer	Parent’s homestead but lives alone
4	Christiana	72	Old and frail, Blind, weak	Brother’s homestead
5	Rebecca	65	Active, but with severe arthritis and unable to move about	Parents homestead; Lives with brother and relatives,
6	Ugonwa	57	Subsistence farmer	Lives alone
7	Regina	46	Petty trader	Lives alone
8	Dinma	66	Subsistence farmer	Lives alone
9	Dorcas	72	Weak and frail	Lives alone in her brother’s homestead
10	Nkechi	68	Sick and frail	Lives in the brother’s homestead
11	Mary	61	Subsistence farmer	Lives alone in isolation

### Data analysis

There are several approaches to analysing narrative accounts (e.g., interactional, performative structural, thematic analyses, see Riessman, 2005). We chose thematic analysis as our focus is on “what” was said rather than “how” it was said. However, as Riessman identifies “Narratives do not speak for themselves or have an unanalysed merit; they require interpretation when used …” (p. 2). We adopted the seven step-method recommended by Colaizzi (1978) in analysing transcripts. Colaizzi recommends: familiarization with the data, identifying significant statements, formulating meanings, clustering themes, developing an exhaustive description, producing a fundamental structure and seeking verification of the fundamental structure as the processes in analysis. Transcripts from the interviews were subjected to iterative reading. As very little is known about the experiences and conditions of childless widows in literature, we adopted an inductive approach in data interpretation. An inductive approach to analysis suggests that themes identified are anchored on the data set (Patton, 2002); thus we were not drawing from any theoretical assumption in examining and analysing the data elicited from the participants. Three of the authors (DU, LU and CO) met severally to harmonize the findings in order to ensure a more appropriate representation of the reports. All the authors personally read, contributed and agreed to a final draft. A final version of the analysis was scripted by CO.

## Results

### The anguish of childless widows

It is important to note that the women interviewed in the current study were involuntarily childless. They desired to have children in line with the norms of their immediate society but could not. Most of them had little or no education. The most educated who incidentally was the youngest of the widows had attained only secondary level education. None of them had engaged in any regular paid work and so received neither pension nor gratuity. A few managed to still do subsistence farming and lived on the proceeds of their farms. There were no government welfare packages to cover any aspect of their lives. Accounts of their life experiences as childless widows showcased prolonged trauma from the loss of their spouses, in many years of their widowhood. Reports of depression and hopelessness, suffering inhumane treatments, deprivation and neglect, isolation from society and community studded their lived experiences. Childlessness spurred more current concerns and was blamed for their vulnerability to abuse and loss. These widows often relied on any available support often coming from the activities of not-for-profit organizations and religious groups that provide relief items and sometimes legal defence for them. However, conditions were still grim for these women. In discussing their experiences, pseudonyms were used to better humanize their lived experiences and also to ensure confidentiality.

#### Complicated/lifelong trauma

The widows often described their lives as a series of sad life events. Though most of them were elderly, and for some, decades have passed since the loss of their husbands, memories of the circumstances surrounding the demise of their spouses were still strong and evoked distressful emotions. More importantly, the demise of their husbands marked the beginning of a turbulent life for each widow. Their widowhood and childlessness often take precedence in their descriptions of their life experiences and they often go ahead to identify other negative life events they had suffered. A complex mix of mental distress including sorrow, confusion, hopelessness, insomnia, soliloquies, self-rejection and despair characterized their narrated experiences. Nwakego, a 90-year-old widow who lived alone and depend on charity narrates how she felt: *“… I am overwhelmed with sorrow, I ask God questions when I am alone, and this world is a hell … My husband is dead, I am childless with four of my siblings all dead*.”. The experience of Ugonwa, 57, a subsistence farmer that also lived alone was similar: *“… My condition disturbs me a lot … no child, husband dead, and I am abandoned by everybody … sleep has eluded me, sometimes, I wander from place to place without any destination*.” These excerpts show how these women put their widowhood and childlessness primary in their life events; they then went ahead to connect other adverse life experiences to the description of their life experiences. These life lots culminate into feelings of depression and self-rejection as can be seen in Nwakego’s narration: “*myself disgusts me*”. Widows also seem to take the blame for all the negative life experiences that they have had. They discuss the shattering of their world and feelings of isolation, abandonment and loneliness.

#### Threatened, dehumanized, and ostracized

As the women grieved their cumulative loses, they recalled inhumane treatments, rejection and alienations meted on them in their communities. Traces of their sufferings are still evident in the current living conditions/experiences of some of them. Nwakego, a 90-year-old widow who lived in isolation recalled how she was exiled from the community: “*They came and broke my door, threw my things outside and asked me to leave … beat me and collected the money the church gave me. Sometimes, they tied me to a tree and disgraced me*.” She now leaves in a separate hut distant from the residential areas of the community. The alienation from society is intensified by the inter-personal clashes the widows had with members of the community in everyday life and the denial of common goods in the community. Nwakego again recalled: “*They do not allow me to take part or share in the things of the village. I buy everything.”* Mgbeke who is 86 years old was forced out of her husband’s house for being childless, but currently resides in her parent’s homestead; she even lives far away from relatives. Mgbeke also her narrated hostile experiences in trying to find a place to settle: “*… I was chased away everywhere I went … even in my father’s house they don’t want me alive. I was poisoned so that the little I have will be taken … they called me a witch*.” Chinasa, 67, who had moved back to her parents’ homestead but lived alone recalled that she was considered a disturbance and nuisance when she tried to participate in the community: “… *in[traditional] social functions and they are sharing goods, if you try to join them, they will tell me to leave that I am disturbing them … I depend on what I produce*.” Christiana, 72, who is blind and lives with her brother who is also elderly described herself to be: “… *a daughter and wife, and therefore have no entitlement except being in my father’s compound*”.

One dehumanizing experience commonly reported by the widows was that they were accused of being witches. One of the widows said: “*Everywhere they call me a witch … that I have eaten up the children in my womb*” (Mgbeke, 86, lives alone). Nwakego also shared another experience: “*Even in the market … they don’t want anything to do with me … they think I will bring them bad luck and see me as a witch that kills people*. Almost always, the degrading experiences of the childless widows begin from the relatives of their late husbands. Regina, 46, whose late husband was an only son and died of leukaemia felt she has lost her autonomy and control: “*… anywhere they asked you to stay, that’s where you stay … they took whatever money and property that was left after the burial rites … tell you what to do and what you will not do* … *you’ll have to obey*”. Regina was the youngest of the widows interviewed and as at the time of the study it was less than a year her husband died. She was pressured by her sisters-in-law to “… *marry a wife in the name of my late husband so that she can raise children to replace their brother and … continue the linage*”. Declining this suggestion for reasons of her religious beliefs Regina reported: “*… how can I marry a wife for a dead person? … I faced trouble from every side because I refused … I decided to leave*”. The fact that Regina’s experience had happened within the past one year demonstrates that [particularly childless] widows continue to suffer abuses and stigmatization from people in their communities. It is even more disturbing that these abuses, as in the case of Regina, are meted out by females.

#### Feelings of abandonment

Ostracized and treated with contempt, widows felt isolated and abandoned. Abandonment re-echoed in the accounts of the widows. They felt left to their own fate; they were not cared for by their families and communities. Christiana, 72 who was blind and lived with her brother, developed an eye problem soon after her husband died and by the end of the burial rites, she was totally blind as no family member agreed to contribute money for a scheduled eye surgery. She gave an account of how abandoned she was: “*There would be food in the house but I could not cook because there is nobody with me. It was only when they returned on Friday that they would cook for me before going back …”* Christiana further narrated how her late husband’s relatives visited once in 15 years: “*… they appeared and said they’ve come to celebrate Christmas … that was 15 years ago nobody has seen them since then*.”

### Childlessness- the crescendo of distress in widowhood

The pangs of widowhood are felt most when childlessness is brought to focus. Having no child was the most distressing concern that continued to have a lifelong impact on the women’s lives. They almost always considered that having [a] child(ren) would have made their lot better.Earlier, we presented Regina’s experience regarding being pressured by sisters-in-law to marry a new wife for her deceased husband so as to preserve their linage. Regina encountered pressures because she had no child[ren]. If she had children, her position among her husband’s relatives would have been better appreciated and she may not have faced so much distress.

In a setting where welfare/social services are not available, livelihood is hard and challenging for these women as many of them are elderly and lived alone. Dinma, 66, who lives alone and was a subsistence farmer, complained of often missing the support of children: *“… after working, I am always hungry and if a child was around, she would have helped to cook something for me to eat … I will have to do everything. Even when I am sick, I am all on my own, until I can find strength to get up and go out, I’ll have to stay alone*”. Dorcas, 72 who lives in her brother’s homestead and is visually impaired was worried about how she’ll cope: “*… I have been going to be treated even to the teaching hospital, but nothing … the eye is painful and I am beginning to have a blurred vision … if anything happens to my sight, hei! I am dead … there’s no child staying with me … nobody to get this and get that for you … how will I manage?*” Dinma’s and Dorcas’s experiences showed how valuable child[ren] are perceived to be by these widows.

Mgbeke, 86, lived alone and depended on charity thought that: “*If I had any child, my situation will not be like this*”. Nwakego, 90, had a particularly remarkable condition as she had no living relative: “*If I had any child or even my siblings’ children, they will stand by me. They will not allow them treat me like that*.” Having children is therefore seen as a guarantee to be accepted by the husband’s relatives. Chinasa, 67, who was a subsistence farmer narrated the challenges she faced with extended family members and how having a child would have made a difference: “… *after my husband died, they started exhibiting bad attitude … they hated me … Had it been I had a child, I would have stayed*.”. Nkechi, 68, who lived in her brother’s homestead described an experience with a widow who had children: “*There was a day a lady whose husband just died came in here and insulted me because of childlessness … she has children … that’s the difference, else we are the same*.” This showed how widows who have children feel better placed and more entitled than childless widows.

### Coping strategies

#### Fitting to other roles

Some widows found livelihood and a sense of motherhood in taking on parenting roles for close relatives, sometimes, amidst ostracism. But this was only limited to widows who lived with their relatives. Chinasa, 67, who was a farmer and lived in her parents’ homestead partook in parenting roles as she groomed her sister’s children: *“… though people caution my elder sister’s daughter that I am not her mother … it is my duty to take care of my elder sister’s child*”. Rebecca, 65, who lived in her parents’ homestead had access to the teeming children and grandchildren of her brother. Living amongst them seemed to ease the pains of being a childless widow as she expressed ownership of the children around her: “*… anyone who says I’m childless is not getting on me … look at all the children around, they take me as their mother*.” Here, the gains of the extended family can be noticed. Rebecca expressed a sense of belonginess and acceptance that countered the cultural stigma of childless widowhood. The motherly role that Rebecca played shielded her from the pangs of childlessness often reported by many other widows. It is important to note that Rebecca’s privilege was uncommon among the widows as most of them lived alone and were perceived to be vicious or diabolical. We learnt from Rebecca’s experience that incorporating these widows into lively homesteads could ease their plights.

#### Resigning to God

Many of the women end the narration of their experiences with a resignation to God. When faced with difficulties and dejection, comments such as “*I handed everything to God*” [Regina, 46, petty trader] and “*I leave them to God*” [Nkechi, 68 & Mary, 61] were noticed throughout the data. Dorcas who lived with her brother’s wife lost hope of getting justice from a local justice system: “*… they refused me farming in a land … I plant, they destroy it … I report [to the irodo- a local justice administrative system] they keep it for long … I look at them and look at my God*.” This resignation to God when these widows felt maltreated underlines the lack of protective structures or institutions that address issues of maltreatment of widows.

#### Support from religious groups and individuals

Many of the women reported that they often survived by the assistance received from religious groups who sought them, did chores for them, and provided consumables. Widows sometimes referred to these religious groups as their only means of survival. Nwakego, 90, who depends on donations from religious charity rhetorically recognized religious groups as her helpers: “*Do I have any child apart from God’s children who use to help me?*” Another identified the protection offered by the intervention of a religious figure in the area in preventing maltreatments “*Father Okeke [not his real name, a popular Catholic priest in the area] came and warned them that they should be mindful of how they treat me … if anything happens to me, he’ll hold them responsible … since then they left me alone*.” Hence religious communities and other local/pressure groups held sway in advocating for and maintaining the welfare of childless widows.

## Discussion

In the current study, we hoped to highlight from the experiences of the widows, that a section of widows, childless widows, may be a more disadvantaged group given sociocultural circumstances. Literature focusing on childless widows are scarce. Our findings from the narratives of childless widows resonate with earlier findings of loneliness, depression and sleep disturbances among widows in general (Nalungwe, 2009). However, there were distinctive experiences as childless widows. Both widowhood and (involuntary) childlessness are considered heavy misfortunes, so much that these widows are blamed for their misfortunes. Thus, there is a strong stigma and segregation meted against this group which impacts their livelihood and wellbeing, at least in the setting studies. Being elderly, having no child, and the long years of difficulty in widowhood made childless widows more devastated and seemingly hopeless. The strong emotional distress found among this category of widows suggests a serious psychological impact spurred by their status and the treatment it attracts within the locale. Their conditions, as reported by the widows can lead them to psychomotor agitation, often found in the accounts of severe depression (Kselman, 2002).

Narratives also revealed persecutions and deprivations meted on this category of women, specifically because of their status as childless widows. These treatments exist regardless of existing government legislations proscribing them. One surprising finding is that some of the inhumane practices perpetrated against this group of widows were championed by other women, and began from their immediate families, as these widows were accused of being witches among other dehumanizing attitudes shown to them. Nayar (2006) reported that deprivation experienced by widows often arise from intra-household and community-based discrimination. These inhumane treatments force the widows to withdraw from their communities. Indeed, many of the widows who shared their experiences were found to be living in isolation. Being childless and widowed, ostracized women begin to lose their sense of belongingness in their communities.

It is very salient that childlessness made widowhood more difficult in the culture and area of focus in this study. Having children in Nigeria, especially in places with poor social welfare systems, is socially desired (Muomah, 2010; Onyishi, Sorokowski, Sorokowska, & Pipitone, 2012). However, we learnt from these childless widows, that the desire to have a child or children transcended social desirability. For the widows, children were a very important resource that provide security and access to traditional rights which would have shielded the widows from harsh treatment in their communities. Similar to many African marriage patterns, marriages among the people of South East Nigeria are often elaborately contracted between families (Foster, 2000). However, in the narration of the childless widows, the bonds between families are totally broken and the women are surprisingly left alone to face severe hardships on both economic and cultural fronts. Milazzo and van de Walle (2018) reported that under some customary laws in Nigeria, childless widows might be asked to leave their matrimonial homes by their in-laws. Also, in Senegal, having sons are very important in insuring women against widowhood (Lambert & Rossi, 2016). Thus, widows are more predisposed to distress when they do not have children.

In African settings, where social welfare schemes are meagre, the elaborate family system is often a formidable social support resource. Nonetheless, childless widows appear to be a specifically isolated group, rejected and ostracized from society and left to suffer psychological and social trauma. Policies targeting to better the lots of widows should be sensitive to identifying that childless widows are a more vulnerable group to be considered.

## Limitations

Similar to many qualitative studies, this study involved few participants who shared personal experiences. Hence, their experiences may only depict personal experiences, or may showcase the actions of a specific culture and people. Our study may therefore not be generalized to all childless widows globally. Nonetheless, studies in Africa (e.g., Milazzo & van de Walle, 2018) are suggestive of similar patterns of treatment for widows who do not have children. In addition, future studies may explore the experiences of childless widows in other settings (especially, other Western and Eastern cultures) to compare and contrast experiences. As all participants resided in a rural community and had very little education, experiences of childless widowhoods may be more different for more educated childless widows residing in urban areas.

## Recommendations and conclusion

Childless widowhoods have substantial distress that possibly transcends widowhood in general. They seem to be more alienated and secluded from society than other widows, especially in low resource regions as demonstrated by the widows in this study. Instead of sympathy and empathy, these set of widows received harsh treatments from relatives, and experienced difficulties in their communities. Cultural beliefs and practices that vilify this segment of widows are still active, despite government laws proscribing them. The narratives of the widows in this study suggest that there is need to provide protective measures for childless widows. The widows often identified religious groups to have played significant roles in supporting them. These groups could be useful in educating communities to become more supportive of the widows. Organizing childless widows into small associations will help them in identifying and addressing their difficulties and collectively pursue their well-being. There is a serious need for social security/welfare systems in Africa and other low resource settings around the globe to create special systems to carter for childless widows. Future research and policies could search to identify and proffer ways to challenge belief systems that uphold dehumanizing practices against widows and particularly, childless widows. Findings from our study will help policy makers and intervention strategists to advocate for and design programmes that will target the specific needs of this neglected segment of the population.
